# Association Between Salt Intake and Body Adiposity in Chinese Population: A Repeated-Measures Cohort Study

**DOI:** 10.3390/nu18060976

**Published:** 2026-03-19

**Authors:** Weiyuan Yao, Xiangyu Chen, Feng Lu, Jie Zhang, Chunxiao Xu, Mingbin Liang, Ruying Hu, Meng Wang, Jieming Zhong, Xiaofu Du

**Affiliations:** Department of Chronic Disease Prevention and Control, Zhejiang Provincial Center for Disease Control and Prevention, Hangzhou 310051, China; ywyao@cdc.zj.cn (W.Y.); xychen@cdc.zj.cn (X.C.); flu@cdc.zj.cn (F.L.); jiezhang@cdc.zj.cn (J.Z.); chxxu@cdc.zj.cn (C.X.); mbliang@cdc.zj.cn (M.L.); ryhu@cdc.zj.cn (R.H.); mwang@cdc.zj.cn (M.W.)

**Keywords:** salt intake, sodium intake, overweight, central obesity, body adiposity, longitudinal study

## Abstract

**Background/Objectives**: Several studies have suggested a positive association between salt intake and obesity, yet longitudinal evidence is limited. We aimed to investigate the longitudinal association between salt intake and multiple adiposity indicators. **Methods**: We used longitudinal data from a cluster-randomized controlled trial conducted in Zhejiang Province, China, including 7372 adults with 12,800 observations. Twenty-four-hour salt intake was estimated using spot urine samples. Adiposity was assessed using body mass index (BMI), body roundness index (BRI), body fat mass, overweight and central obesity. Associations between salt intake and adiposity were evaluated using generalized linear mixed-effects models. Mediation analyses were conducted to quantify the proportion of associations mediated by body fat mass. **Results**: Mean baseline 24 h salt intake was 9.88 g/d. Compared with participants in the lowest quartile of salt intake (<8.4 g/d), those in the highest quartile (≥11.2 g/d) had higher BMI (difference, 1.14 kg/m^2^; 95%CI, 1.03–1.25), BRI (0.31, 0.26–0.35), body fat mass (1.88 kg, 1.69–2.07), and higher odds of overweight (OR, 2.82; 95%CI, 2.47–3.22) and central obesity (2.78, 2.42–3.20). Longitudinally, reductions in salt intake (>1 g/d) were associated with decreases in BMI [−0.21 kg/m^2^ (−0.33, −0.09)], BRI [−0.04 (−0.09, 0.00)], and body fat mass [−0.14 kg (−0.36, 0.07)]. Associations were stronger among women and adults aged < 40 years (*p*-values < 0.05). Body fat mass mediated 56.93%–84.73% of the associations. **Conclusions**: This study indicates a dose–response association between salt intake and obesity risk, partly mediated by increased body fat mass. The findings suggest that dietary salt may influence cardiovascular risk through adiposity-related pathways.

## 1. Introduction

The 2021 Global Burden of Disease Study estimated that 2.11 billion adults around the world were overweight or obese, accounting for 45.1% of the global adult population [1]. Obesity is not only a disease but also a major risk factor for cardiovascular diseases, metabolic diseases, and cancers, making it a critical public health crisis that demands urgent measurements [2]. Well-established determinants of obesity include physical inactivity, excessive energy intake, genetic predisposition, and psychological factors [3]. Emerging evidence suggests that salt (sodium) intake is also associated with body weight and adiposity, indicating that salt reduction may contribute to obesity prevention [3,4,5,6], though evidence for a direct weight-loss effect remains limited. Animal studies had shown that high-salt diets can activate the aldose reductase–fructokinase pathway in the liver and hypothalamus, increasing the risk of obesity, insulin resistance, and fatty liver disease [7].

However, the findings from human studies are controversial. Meta-analyses of cross-sectional studies reported positive associations between sodium intake and overweight or central obesity after adjustment for energy intake and lifestyle factors [3,8,9]. Previous randomized controlled trials (RCTs) of sodium reduction reported no significant effects on body weight [8,10,11,12]. However, most were designed to evaluate blood pressure rather than adiposity and therefore may have been underpowered to detect differences in body weight or fat mass. To date, only one RCT (*n* = 85) assessed adiposity specifically and found that weight reduction in the low-salt-diet group was attributable to loss of body water rather than fat mass [13]. Conversely, a longitudinal study (*n* = 215) reported that higher 24 h urinary sodium excretion was associated with increased fat mass and decreased fat-free mass, but not with changes in weight or waist circumference [14]. These studies were limited by small sample size [13] or by the lack of repeated measurements of sodium intake [14], leaving important gaps regarding the association between salt intake and obesity and the underlying mechanisms.

Body mass index (BMI), the most commonly used anthropometric measure, cannot adequately capture whole-body fat distribution or differentiate fat mass from lean mass [15]. Recently, several new indices were developed to provide more comprehensive understanding of body composition and its relationship with health status. The body roundness index (BRI), an index calculated from waist circumference relative to height to quantify body shape independent of body height [16], demonstrated better predictive ability for metabolic syndrome than BMI [17].

In this study, using longitudinal data from the Salt Reduction and Hypertension Prevention Project (SRHPP) in Zhejiang province, China, we aimed to (1) examine the association between salt intake and multiple indicators of adiposity, including BMI, BRI, body fat mass, and waist circumference; and (2) assess whether sodium intake influences obesity risk through increases in body fat mass.

## 2. Materials and Methods

### 2.1. Study Design and Participants

This study was a secondary analysis of the SRHPP, a cluster-randomized controlled trial conducted in Zhejiang Province, China (Registration: ChiCTR2000033349, China Clinical Trial Registry). The trial was designed to evaluate the effectiveness of comprehensive salt reduction and hypertension prevention strategies on blood pressure control and related health outcomes in a Chinese population. Details of the trial have been published previously [18,19].

Briefly, a multistage sampling approach was used to recruit 7512 participants from five counties between December 2016 and May 2017. The five counties were selected from Zhejiang Province to ensure representativeness, followed by proportional probability sampling of five towns or streets within each county and three villages or communities within each sampled town or street. From each selected village or community, 100 adults aged 18–69 years were randomly selected and invited to participate in face-to-face interviews and physical examinations. Inclusion criteria required participants to be aged 18–69 years, willing to participate, free from physical disabilities or mental disorders, and living in the selected areas for at least six months. Randomization was then performed at the county level following the baseline survey. A total of 6003 participants from four counties were assigned to receive two years of comprehensive health education on salt reduction and hypertension prevention, while the remaining 1509 participants from another county served as the control group received no intervention. After a two-year intervention and an additional two-year post-intervention follow-up, 6010 participants were resurveyed in December 2021. At both baseline and follow-up, trained health workers conducted face-to-face questionnaires, physical examinations, and blood and urine tests.

For the present analysis, we initially included 7390 participants with complete data from questionnaires, physical examinations, blood tests, and spot urine tests in the baseline survey, with 5717 participants having complete data at follow-up. After excluding outliers in anthropometric and laboratory measures, 7276 participants remained at baseline and 5524 at follow-up. The final analytical cohort comprised 7372 participants with 12,800 observations, of whom 5428 had complete data at both baseline and follow-up (Supplementary Figure S1).

The SRHPP study protocol was approved by the Ethics Review Committee of the Zhejiang Provincial Center for Disease Control and Prevention (protocol code: 2019056 and date: 30 December 2019). Informed consent was obtained from all participants.

### 2.2. Estimation of 24 h Salt Intake

Fasting morning urine samples were collected from all participants in both surveys. At baseline, 1424 randomly selected participants provided 24 h urine samples following a standard collection protocol. Using the data of these paired-spot and 24 h urine samples, we previously developed the Zhejiang model to predict 24 h urinary sodium excretion (24 hUNa_pre_) from fasting morning urine biomarkers [19]. This model demonstrated superior predictive performance compared with the Tanaka and INTERSALT model in the Chinese population [20,21].

In the present study, 24 h urinary sodium excretion for all participants was estimated using the Zhejiang Model (Equations (1) and (2)). The estimated 24 h salt intake was then derived using Equation (3).

24 hUNa_pre_ for males = (0.69 × Na + 42.54) − 2.98 × Cr + 0.02 × K + 0.14 × Ma + 0.31 × height + 0.66 × weight + 1.08 × age − 0.03 × age^2^ (1)

24 hUNa_pre_ for females = (0.43 × Na − 131.88) − 2.86 × Cr + 0.21 × K + 0.08 × Ma + 1.02 × height + 0.86 × weight + 3.85 × age − 0.05 × age^2^ (2)

Estimated 24 h salt intake = 24 hUNa_pre_ × 0.0585 (3)

Na: spot urinary sodium (mmol/L), K: spot urinary potassium (mmol/L), Cr: spot urinary creatinine (mmol/L), Ma: spot urinary microalbumin (mg/L). The units of weight, height, age, 24 hUNa_pre_, and 24 h salt intake were kg, cm, year, mmol/24 h, and g/d, respectively.

### 2.3. Anthropometric Measurements

At each survey, anthropometric data were collected by trained health workers following standard procedures. Height (cm), weight (kg), and waist circumference (cm) without shoes or heavy clothing were measured to the nearest 0.1 unit. Body fat mass (kg) was assessed using the bio-impedance method (OMRON HBF-371). BMI and BRI were calculated using the following formulas:

1. $BMI=weight(kg)/{height}^{2}(m)$
2. $BRI=364.2-365.5\times \sqrt{1-\frac{{\left({waist\;circumference}_{\left(m\right)}/2\pi \right)}^{2}}{{\left(0.5*{height}_{\left(m\right)}\right)}^{2}}}$ [16]

Overweight was defined as BMI ≥ 24 kg/m^2^, and central obesity was defined as WC ≥ 85 cm for women and ≥90 cm for men [22].

### 2.4. Covariates

The covariates considered in this analysis included age, sex, educational level, residential area, alcohol drinking status, smoking status, physical exercise, dietary preferences, history of hypertension, history of diabetes, and laboratory measurements. Educational level was categorized as low (primary school or below), medium (junior and senior high school), or high (college or higher). Residential area was classified as rural or urban. Alcohol drinking status was defined as yes or no, while smoking status were categorized as never, former, or current smoker. Physical exercise (yes or no) was defined based on the question “Do you engage in leisure-time physical activities during a typical week?”. Dietary preferences were assessed using three questions about participants’ usual diet compared to their peers, which were used to generate binary variables (yes or no) for preference for salty foods, oily foods, and meat-based diets. History of hypertension (yes or no) was defined as self-reported physician diagnosis, current use of antihypertensive medication, or measured systolic blood pressure ≥ 140 mmHg or diastolic blood pressure ≥ 90 mmHg [23]. History of diabetes was defined as self-reported physician diagnosis, current use of antidiabetic medication, or fasting blood glucose ≥ 7.0 mmol/L [24]. Laboratory measurements included total cholesterol (TC), triglyceride (TG), high-density lipoprotein cholesterol (HDL-c), and low-density lipoprotein cholesterol (LDL-c).

### 2.5. Statistical Analysis

Baseline characteristics of participants were summarized as means (standard deviation) for continuous variables and frequencies (percentage) for categorical variables. Comparisons across groups defined by 24 h salt intake levels were preformed using analysis of variance, Kruskal–Wallis tests, or chi-square tests, as appropriate.

We first applied mixed-effects models to assess the associations between 24 h salt intake and adiposity indices incorporating individual-specific random intercepts, accounting for repeated measurements (level-1) nested within individuals (level-2). Restricted cubic splines (RCS) with three knots and likelihood ratio tests were performed to explore potential non-linear relationships between salt intake and adiposity [25]. Potential confounders were considered in these models, including age, sex, educational level, residential area, alcohol drinking status, smoking status, physical exercise, dietary preferences, TC, TG, HDL-c, LDL-c, history of hypertension, and history of diabetes (Supplementary Table S1).

Linear and monotonic relationships between 24 h salt intake and BMI, BRI, and body fat mass were observed. Consequently, all participants were classified into four groups based on quartiles of 24 h salt intake. Linear mixed-effects models (LMMs) were used to estimate regression coefficients with 95% confidence intervals (CIs) for differences in BMI, BRI, and body fat mass across groups, adjusting for potential confounders. Generalized linear mixed-effects models (GLMMs) were applied to calculate the odds ratios (ORs) with 95%CIs for the associations between 24 h salt intake and risks of overweight and central obesity.

Second, we evaluated the longitudinal association between 24 h salt intake and adiposity indices. Participants with complete data at both baseline and follow-up surveys were reclassified into three groups: stable (change in 24 h salt intake < 1 g between baseline and follow-up), decrease (follow-up 24 h salt intake was 1 g lower than baseline), and increase (follow-up 24 h salt intake was 1 g higher than baseline). Multivariable linear models were used to assess the mean differences in BMI, BRI, and body fat mass at follow-up across groups. The ORs with 95%CIs for follow-up overweight and central obesity status for decrease and increase groups (vs. the stable group) were also estimated. All longitudinal models were adjusted for potential confounders and corresponding baseline adiposity measures.

Moreover, mediation analyses were conducted to examine whether body fat mass mediated the association between salt intake and adiposity using longitudinal data. There was no interaction between salt intake and body fat mass on the associations with adiposity indices. Therefore, the total effect of salt intake on obesity was considered the sum of the directed effect and the mediated effect. Bootstrapped CIs for mediation analysis were obtained using 1000 resamples. Stratified analyses were conducted to evaluate the potential effect modification by sex, age, and history of hypertension or diabetes.

All statistical analyses were conducted using SAS version 9.4 (SAS Institute Inc., Cary, CA, USA) and R version 4.3 (R Foundation for Statistical Computing, Vienna, Austria). All tests were two-sided, and *p* values < 0.05 were considered statistically significant.

## 3. Results

### 3.1. Baseline Characteristics of Study Participants

Table 1 presents the baseline characteristics of the 7276 participants. The mean estimated 24 h salt intake at baseline was 9.88 g/d, which exceeded the national recommendation for Chinese adults during most of the study period (≤6 g/d) and the current recommendation (<5 g/d). Participants were classified into quartiles of salt intake based on the distribution in the study population, with cut-off points at 8.4, 9.8, and 11.2 g/d. Across increasing quartiles of salt intake, participants tended to be younger, have higher educational attainment, and report a preference for salty foods. Significant differences across groups were also observed for sex, alcohol drinking, smoking status, preference for oily or meat-based foods, hypertension, TG, and HDL-c levels. No significant differences were observed for residential area, exercise, diabetes, TC, LDL-c, or fasting blood glucose (all *p* > 0.05).

### 3.2. Association Between Salt Intake and Adiposity Indices

**Figure 1:**
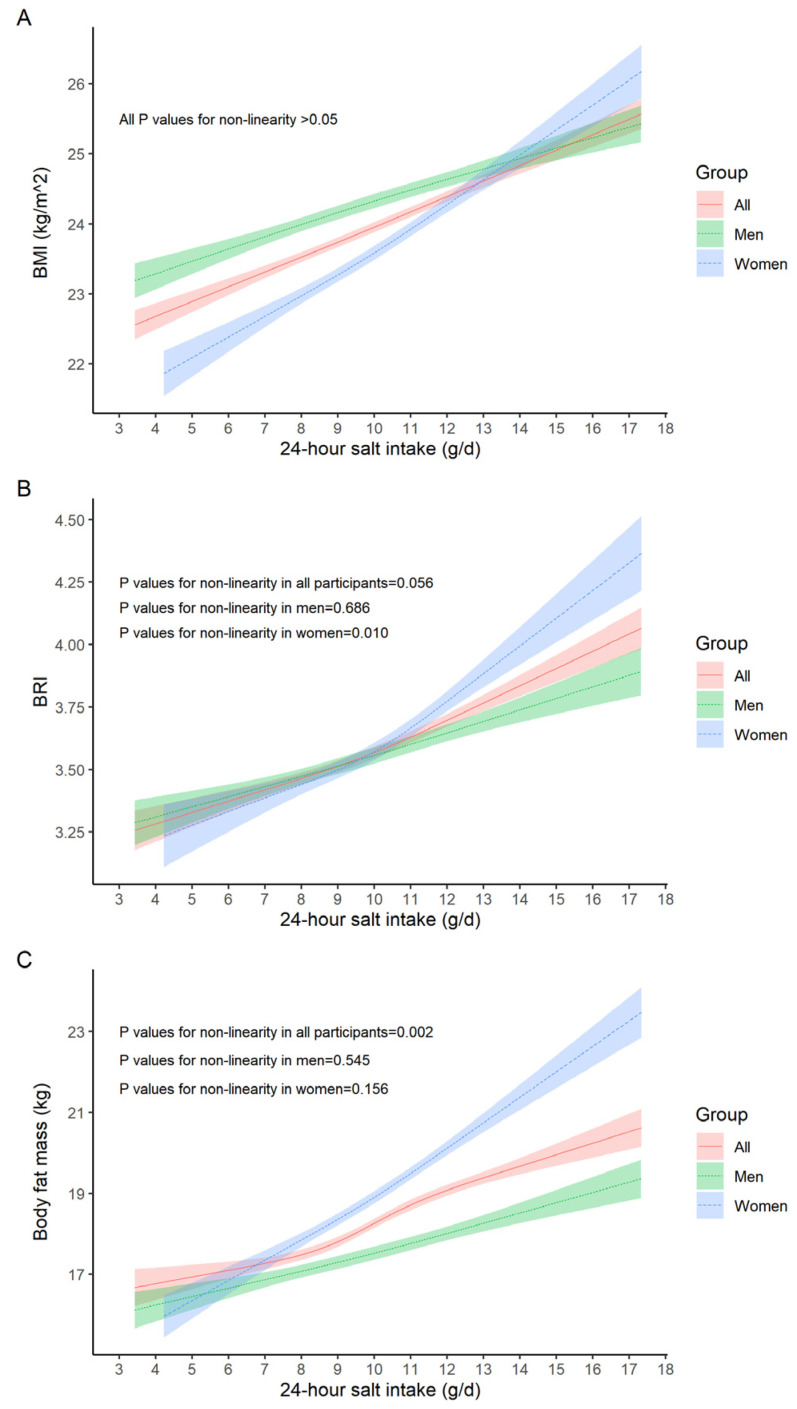
Relationship of 24 h salt intake with BMI, BRI, and body fat mass by sex. (**A**) The association between 24 h salt intake with BMI; (**B**) the association between 24 h salt intake with BRI; (**C**) the association between 24 h salt intake with body fat mass. The 24 h salt intake was calculated based on the 24 h urinary Na excretion estimated using spot urine sample with the Zhejiang model. The curves were fitted by mixed-effects models with fixed effect given by a cubic spline for 24 h salt intake (g/d) and with participant-specific random intercept, adjusted for sex, living area, educational level, physical exercise, smoking status, alcohol drinking, and dietary preferences, total cholesterol, triglyceride, high-density lipoprotein cholesterol, low-density lipoprotein cholesterol, fasting blood glucose, hypertension, and diabetes.

As shown in Figure 1, BMI, BRI, and body fat mass increased monotonically with 24 h salt intake. The rate of increase was higher in women (all *p*-values for sex interaction < 0.05). BRI increased more rapidly at salt intake levels exceeding 11 g/d among women, while body fat mass showed a steeper increase at salt intake levels above 9 g/d in all participants (both *p*-values for nonlinearity < 0.05).

The age- and sex-adjusted means of BMI, BRI, and body fat mass were significantly higher among participants with greater salt intake compared with the lowest quartile (Table 2). After adjusting for potential confounders, a 1 g/d increase in salt intake was associated with 0.22 kg/m^2^ higher BMI (95%CI: 0.20, 0.23), 0.06 higher BRI (95%CI: 0.05, 0.07), and 0.34 kg higher body fat mass (95%CI: 0.31, 0.38). Compared with participants consuming < 8.4 g/d, those in higher salt intake quartiles (8.4–9.8 g/d, 9.8–11.2 g/d, and ≥11.2 g/d) had higher BMI [differences: 0.31 (95%CI: 0.21, 0.41), 0.69 (0.59, 0.80), 1.14 (1.03, 1.25) kg/m^2^], BRI [differences: 0.08 (0.04, 0.12), 0.19 (0.14, 0.23), 0.31 (0.26, 0.35)], and body fat mass [differences: 0.48 (0.31, 0.66), 1.09 (0.91, 1.28), 1.88 (1.69, 2.07) kg]. A significant sex heterogeneity was found in the associations between salt intake and adiposity indices, with stronger associations observed among women than men (*p*-values for sex interaction < 0.05).

**Figure 2:**
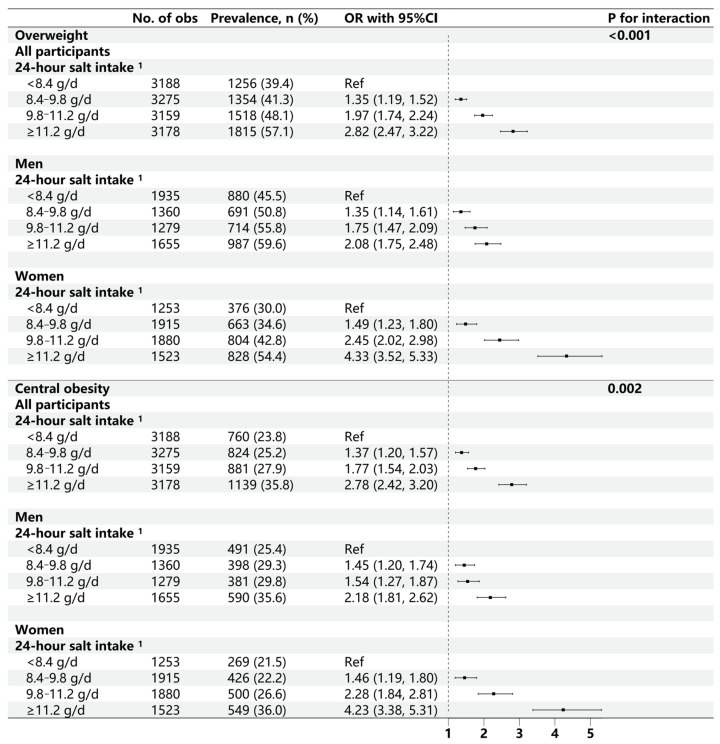
Association between 24 h salt intake and the risk of overweight and central obesity by sex. ^1^ The 24 h salt intake was calculated based on the 24 h urinary Na excretion estimated using spot urine sample with the Zhejiang model. No. of obs: available observations of the eligible participants. The OR with 95%CI estimated using generalized linear mixed-effects models adjusted for age, sex, living area, educational level, physical exercise, smoking status, alcohol drinking, and dietary preferences, total cholesterol, triglyceride, high-density lipoprotein cholesterol, low-density lipoprotein cholesterol, fasting blood glucose, hypertension, and diabetes. *p* values: p for interaction between 24 h salt intake and sex.

A higher prevalence of overweight and central obesity was observed among participants with higher estimated 24 h salt intake (Figure 2). Multivariable models showed that a 1 g/d higher salt intake was associated with 21% higher odds of overweight (95%CI: 1.19, 1.24) and 20% higher odds of central obesity (95%CI: 1.17, 1.23). Compared to participants in the lowest quartile of salt intake, higher odds of overweight [ORs: 1.35 (1.19, 1.52), 1.97 (1.74, 2.24), 2.82 (2.47, 3.22)] and central obesity [ORs: 1.37 (1.20, 1.57), 1.77 (1.51, 2.03), 2.78 (2.42, 3.20)] across quartiles were observed. Stratified analyses demonstrated stronger associations in women than men (all *p*-values for sex interactions < 0.05).

### 3.3. Longitudinal Association Between Salt Intake and Adiposity Indices

**Figure 3:**
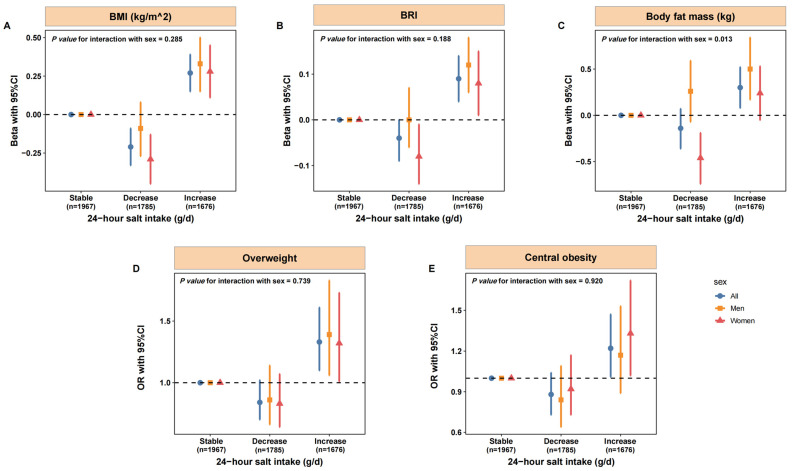
Longitudinal association between 24 h salt intake and measures of adiposity over a 4-year period. (**A**) The longitudinal association between 24 h salt intake and BMI; (**B**) The longitudinal association between 24 h salt intake and BRI; (**C**) The longitudinal association between 24 h salt intake and body fat mass; (**D**) The longitudinal association between 24 h salt intake and risk of overweight; (**E**) The longitudinal association between 24 h salt intake and risk of central obesity. The 24 h salt intake was calculated based on the 24 h urinary Na excretion estimated using spot urine sample with the Zhejiang model. Participants were divided into three groups: stable (change in 24 h salt intake < 1 g between baseline and follow-up), decrease (follow-up 24 h salt intake was 1 g lower than baseline), and increase (follow-up 24 h salt intake was 1 g higher than baseline). n: number of eligible subjects participated in both baseline and follow-up survey. The beta with 95%CI estimated using linear models adjusted for baseline age, sex, living area, educational level, physical exercise, smoking status, alcohol drinking, and dietary preferences, total cholesterol, triglyceride, high-density lipoprotein cholesterol, low-density lipoprotein cholesterol, fasting blood glucose, hypertension, diabetes, and baseline body measurements. The OR with 95%CI estimated using generalized linear models adjusted for baseline age, sex, living area, educational level, physical exercise, smoking status, alcohol drinking, and dietary preferences, total cholesterol, triglyceride, high-density lipoprotein cholesterol, low-density lipoprotein cholesterol, fasting blood glucose, hypertension, diabetes, and baseline body measurements. P for interaction between 24 h salt intake group and sex.

Participants with increased salt intake over four years had greater BMI [0.27 kg/m^2^ (0.15, 0.39)], BRI [0.09 (0.04, 0.14)], and body fat mass [0.30 kg (0.08, 0.52)], compared with individuals whose salt intake remained stable (Figure 3). Increased salt intake was also associated with higher risks of overweight [OR: 1.33 (1.10, 1.61)] and central obesity [1.22 (1.01, 1.47)]. Moreover, participants with decreased salt intake had reductions in BMI [−0.21 kg/m^2^ (−0.33, −0.09)], BRI [−0.04 (−0.09, 0.00)], and body fat mass [−0.14 kg (−0.36, 0.07)]. Stratified analyses by sex showed more pronounced association among women, with −0.46 kg (−0.74, −0.19) lower body fat mass among women in the decrease group (*p*-value for interaction = 0.013).

### 3.4. Mediating Role of Body Fat Mass

Mediation analyses indicated that body fat mass significantly mediated the association between salt intake and adiposity measures (Table 3). The estimated proportion of the total effect mediated by body fat mass was 56.93% (95%CI: 47.96, 66.73) for BMI and 78.57% (58.90, 111.20) for BRI. Additionally, body fat mass mediated 63.29% (50.38, 83.26) of the association between salt intake and the risk of overweight, and 84.73% (56.17, 171.90) of the association with central obesity.

### 3.5. Stratified Analyses

A significant effect modification by age was observed, with stronger associations between salt intake and BMI, BRI, body fat mass, and central obesity among participants aged < 40 years (all *p*-values for age interaction < 0.05, Supplementary Tables S2 and S3). Longitudinal analyses by age group confirmed more evident associations between increased salt intake and adiposity in younger individuals (*p*-values for interaction < 0.05, Supplementary Tables S4 and S5). However, a significantly negative association between decreased salt intake and body fat mass was observed among participants aged < 30 years or >60 years.

Stratified analyses by hypertension and diabetes status revealed consistent positive associations between salt intake and obesity measures among participants without these diseases, while decreased salt intake was correlated with lower BMI and reduced risk of overweight among those with hypertension (Supplementary Tables S6–S9). Both pooled and longitudinal analyses demonstrated that disease status significantly modified the association between salt intake and body fat mass, with stronger association among participants without hypertension and diabetes (all *p*-values for interaction < 0.05).

## 4. Discussion

Using longitudinal data from a large cluster-randomized controlled trial in Zhejiang Province, China, we found that higher salt intake was significantly associated with increased BMI, BRI, body fat mass, and elevated risks of overweight and central obesity. These associations were stronger among women and adults aged < 40 years. We also identified body fat mass as a partial mediator of the salt–adiposity relationship. Collectively, these findings provided crucial epidemiological evidence that excessive salt intake may contribute to obesity partly through increases in body fat accumulation.

The positive associations of salt intake with adiposity indices are consistent with evidence from previous cross-sectional studies [4,5,6,26]. It is of note that the 0.22 kg/m^2^ increases in BMI per 1 g/d higher salt intake observed in our study were lower than the 1.49 kg/m^2^ reported in a smaller NHANES analysis (*n* = 730) [4], while higher than the 0.10 kg/m^2^ increase found in a sample of 839 Chinese participants in the INTERMAP study [5]. Moreover, we observed 2.82-fold higher odds of overweight among participants in the highest quartile versus lowest quartile of salt intake, which was higher than the 1.93-fold increase observed in the NHANES study [4], yet lower than the 4.30-fold increase reported in Finnish adults [6]. A Korean study including 16,250 adults reported higher odds of overweight (2.17-fold) and central obesity (2.50-fold) among adults with sodium excretion ≥ 3200 mg compared to those with sodium excretion < 2200 mg [26]. The heterogeneity between studies may attribute to the difference in study population, sample size and methods used to estimate salt intake.

There are only few prospective studies that have assessed the relationship between salt intake and body adiposity. A cohort study of 215 Danish adults reported no association between sodium intake and body weight or waist circumference, while observing a modest increase in body fat associated with higher salt intake [14]. However, the sodium excretion was measured only once at baseline, potentially missing the within-person change over time. A RCT of 85 adults found reduction in body weight, BMI, and body water following low-salt intervention, while reporting no significant change in waist circumference and body fat mass, possibly due to insufficient statistical power [13]. By contract, our longitudinal analysis, based on repeated measurements from more than 5400 participants, demonstrated clear positive associations between changes in salt intake and risk of obesity after adjusting for potential confounders. These findings strengthen the evidence that salt intake may contribute to obesity development.

Moreover, we observed significant effect modification by sex and age, with stronger salt–obesity associations among women and younger adults. The sex heterogeneity aligns with NHANES findings (*n* = 9162) showing significant associations between salt intake and risk of obesity and abdominal obesity only among women [27], although several other studies reported no interaction by sex [4,14]. In addition, a national survey conducted in South Korea observed a stronger salt–obesity association among teenagers (*n* = 1476, OR with 95%CI: 5.80, 3.17–10.60) than adults (*n* = 16,250, OR with 95%CI: 2.17, 1.90–2.49) [26], whereas a UK study observed comparable association in children (*n* = 458) and adults (*n* = 785) [28]. These discrepancies may potentially be related to sample size. Our study included 7372 participants with 12,800 observations, which had enough statistical power to detect effect modifications.

The underlying mechanisms of the salt–obesity relationship remain unclear. One hypothesis is that high salt intake co-occurs with the consumption of processed energy-dense food or increases intake of sugar-sweetened beverages driven by thirst [8]. However, previous studies observed robust association between salt intake and adiposity independent of energy intake or beverage consumption [4,5,28]. Our results similarly showed that adjustment for multiple metabolic and lifestyle factors did not attenuate the associations. Another possible mechanism was proposed based on animal experiments. High salt intake may activate the aldose reductase–fructokinase pathway in the liver and hypothalamus, promoting endogenous fructose production with the development of leptin resistance and hyperphagia, causing obesity, insulin resistance, and fatty liver disease [7,29]. The observed increased body fat mass related to higher salt intake and the mediating role of body fat mass in the salt–obesity association supported this hypothesis. Future studies are needed to validate these pathways in human populations.

High sodium consumption not only elevates blood pressure [30,31], but may also exacerbate the deleterious effects of obesity [32], increasing the risk of cardiovascular and cerebrovascular events, contributing to an estimated 1.9 million deaths annually [33]. Despite global efforts, mean sodium intake remains high at approximately 4.3 g/d (equivalent to about 10.8 g/d of salt), more than double the recommendation of 2 g/d sodium (5 g/d of salt), and the progress toward the global target of a 30% reduction by 2030 has been slow [34]. Our findings on the salt–obesity link may further heighten public awareness of the health risks associated with high-salt diets, increasing motivation for adopting low-salt diet.

This study benefits from several strengths. First, the large sample size allowed us to precisely evaluate the associations, explore the potential mediating role of body fat, and examine the possible effect modifications. Second, we performed both pooled cross-sectional and longitudinal analysis based on a prospective cohort, using linear mixed-effects models and adjusting for potential confounders, which provided convictive epidemiological evidence for the salt–obesity relationship. Finally, we further found the mediating effect of the body fat mass on the association between salt intake and adiposity indices. The finding promotes understanding of the underlying pathways linking salt intake with obesity.

There are also some limitations that should be cautious. First, salt intake was estimated using spot urine sample, which may generate nondifferential misclassification bias. Although 24 h urine collection is the gold standard, it is impractical for large-scale longitudinal surveys. In this study, we applied the population-specific Zhejiang model, with a superior performance than other models [19], which could provide a relatively solid estimation on salt intake. Second, we were unable to adjust for energy intake due to lack of detailed dietary data, raising possibility for residual confounding. However, adjustment for fasting blood glucose, lipid profiles, and other energy-related covariates did not substantially change the results (Supplementary Table S1). Previous studies similarly suggested a persistent salt–obesity relationship after adjusting for energy intake, which further alleviates our concern. In addition, physical exercise was assessed using a binary variable (yes/no), and information on frequency, duration, and intensity was unavailable, which may have resulted in residual confounding.

## 5. Conclusions

Our findings indicate that salt intake is not only a concern for hypertension prevention, but may also play a meaningful role in the development of obesity. In this large Chinese cohort, salt intake was positively associated with adiposity and risks of overweight and central obesity, with body fat mass partly explaining these relationships. These results highlight salt reduction as a potential public health intervention that could help curb both obesity and cardiometabolic risk. Further studies are warranted to confirm our results and elucidate underlying biological mechanisms.

## Figures and Tables

**Table 1:** Baseline characteristics of the participants according to the estimated 24 h salt intake.

	Level of 24 h Salt Intake ^1^				*p* Values ^2^
	<8.4 g/d (N = 1739)	8.4–9.8 g/d (N = 1922)	9.8–11.2 g/d (N = 1815)	≥11.2 g/d (N = 1800)	
Age (year)	49.63 ± 15.09	45.8 ± 14.15	43.86 ± 12.91	40.54 ± 12.1	<0.001
Sex					<0.001
Men	1101 (63.3)	810 (42.1)	755 (41.6)	955 (53.1)	
Women	638 (36.7)	1112 (57.9)	1060 (58.4)	845 (46.9)	
Residential area					0.079
Urban	794 (45.7)	838 (43.6)	823 (45.3)	753 (41.8)	
Rural	945 (54.3)	1084 (56.4)	992 (54.7)	1047 (58.2)	
Educational level					<0.001
Primary school or below	638 (36.7)	607 (31.6)	524 (28.9)	444 (24.7)	
Junior and senior high school	796 (45.8)	926 (48.2)	877 (48.3)	907 (50.4)	
College or higher	305 (17.5)	389 (20.2)	414 (22.8)	449 (24.9)	
Alcohol drinking	600 (34.5)	603 (31.4)	563 (31.0)	646 (35.9)	0.003
Smoking status					<0.001
Never	1034 (59.5)	1453 (75.6)	1398 (77.0)	1311 (72.8)	
Former	111 (6.4)	72 (3.7)	65 (3.6)	56 (3.1)	
Current	594 (34.2)	397 (20.7)	352 (19.4)	433 (24.1)	
Physical exercise	682 (39.2)	787 (40.9)	741 (40.8)	715 (39.7)	0.0657
Dietary preferences					
Salty	338 (19.4)	389 (20.2)	415 (22.9)	431 (23.9)	<0.001
Oily	322 (18.5)	338 (17.6)	318 (17.5)	349 (19.4)	<0.001
Meat dish	197 (11.3)	168 (8.7)	158 (8.7)	219 (12.2)	<0.001
Hypertension	713 (41.0)	667 (34.7)	590 (32.5)	596 (33.1)	<0.001
Diabetes	143 (8.2)	140 (7.3)	124 (6.8)	134 (7.4)	0.459
TC (mmol/L)	4.92 ± 0.97	4.99 ± 0.97	4.96 ± 0.95	5.00 ± 0.99	0.112
TG (mmol/L)	1.49 ± 1.17	1.47 ± 1.23	1.45 ± 1.25	1.61 ± 1.25	<0.001
HDL-C (mmol/L)	1.27 ± 0.32	1.30 ± 0.31	1.31 ± 0.32	1.27 ± 0.32	<0.001
LDL-C (mmol/L)	2.70 ± 0.76	2.73 ± 0.78	2.72 ± 0.75	2.74 ± 0.78	0.405
Fasting blood glucose (mmol/L)	5.15 ± 1.18	5.16 ± 1.31	5.12 ± 1.27	5.15 ± 1.36	0.105

Data presented as mean ± standard deviation for continuous variables or *n* (%) for categorical variables. Abbreviations: N, number of participants; TC, total cholesterol; TG, triglyceride; HDL-C, high-density lipoprotein cholesterol; LDL-C: low-density lipoprotein cholesterol. ^1^ The 24 h salt intake was calculated based on the 24 h urinary Na excretion estimated using a spot urine sample with the Zhejiang model. Participants were then stratified into quartiles based on 24 h salt intake levels, categorized as Q1 (<8.4 g/d), Q2 (8.4 to <9.8 g/d), Q3 (9.8 to <11.2 g/d), and Q4 (≥11.2 g/d). ^2^ *p* values for the differences among groups using analysis of variance or Kruskal–Wallis tests (continuous variables) or chi-square tests (categorical variables).

**Table 2:** Association between 24 h salt intake and measures of adiposity by sex.

	All Participants			Men			Women			*p* Values
	No. of Obs	Mean (95%CI)	β with 95%CI	No. of Obs	Mean (95%CI)	β with 95%CI	No. of Obs	Mean (95%CI)	β with 95%CI	
Body mass index (kg/m^2^)										
24 h salt intake ^1^										<0.001
<8.4 g/d	3188	22.81 (22.70, 22.92)	Ref.	1935	23.52 (23.38, 23.67)	Ref.	1253	22.03 (21.86, 22.20)	Ref.	
8.4–9.8 g/d	3275	23.57 (23.46, 23.68) *	0.31 (0.21, 0.41)	1360	24.14 (23.97, 24.30) *	0.28 (0.15, 0.41)	1915	23.02 (22.88, 23.15) *	0.42 (0.27, 0.57)	
9.8–11.2 g/d	3159	24.25 (24.14, 24.36) *	0.69 (0.59, 0.80)	1279	24.58 (24.41, 24.75) *	0.54 (0.39, 0.68)	1880	23.90 (23.77, 24.04) *	0.92 (0.76, 1.08)	
≥11.2 g/d	3178	25.18 (25.07, 25.29) *	1.14 (1.03, 1.25)	1655	25.15 (25.00, 25.31) *	0.85 (0.71, 0.99)	1523	25.20 (25.04, 25.35) *	1.54 (1.36, 1.71)	
Body roundness index										
24 h salt intake ^1^										0.074
<8.4 g/d	3188	3.31 (3.27, 3.35)	Ref.	1935	3.35 (3.30, 3.40)	Ref.	1253	3.28 (3.22, 3.34)	Ref.	
8.4–9.8 g/d	3275	3.48 (3.45, 3.52) *	0.08 (0.04, 0.12)	1360	3.51 (3.46, 3.57) *	0.09 (0.04, 0.14)	1915	3.45 (3.40, 3.50) *	0.09 (0.03, 0.14)	
9.8–11.2 g/d	3159	3.64 (3.60, 3.67) *	0.19 (0.14, 0.23)	1279	3.63 (3.57, 3.68) *	0.16 (0.11, 0.21)	1880	3.65 (3.60, 3.69) *	0.22 (0.16, 0.28)	
≥11.2 g/d	3178	3.86 (3.82, 3.90) *	0.31 (0.26, 0.35)	1655	3.76 (3.72, 3.81) *	0.25 (0.19, 0.30)	1523	3.95 (3.89, 4.00) *	0.38 (0.31, 0.45)	
Body fat mass (kg)										
24 h salt intake ^1^										<0.001
<8.4 g/d	3188	16.51 (16.34, 16.69)	Ref.	1935	16.39 (16.16, 16.63)	Ref.	1253	16.49 (16.23, 16.75)	Ref.	
8.4–9.8 g/d	3275	17.56 (17.39, 17.72) *	0.48 (0.31, 0.66)	1360	17.15 (16.88, 17.42) *	0.43 (0.17, 0.68)	1915	17.98 (17.78, 18.19) *	0.69 (0.45, 0.93)	
9.8–11.2 g/d	3159	18.72 (18.55, 18.89) *	1.09 (0.91, 1.28)	1279	17.94 (17.66, 18.21) *	0.81 (0.54, 1.07)	1880	19.47 (19.26, 19.67) *	1.52 (1.26, 1.77)	
≥11.2 g/d	3178	20.12 (19.95, 20.29) *	1.88 (1.69, 2.07)	1655	18.82 (18.57, 19.07) *	1.32 (1.06, 1.59)	1523	21.40 (21.17, 21.63) *	2.62 (2.34, 2.90)	

^1^ The 24 h salt intake was calculated based on the 24 h urinary Na excretion estimated using spot urine sample with the Zhejiang model. Participants were then stratified into quartiles based on 24 h salt intake levels, categorized as Q1(<8.4 g/d), Q2 (8.4 to <9.8 g/d), Q3 (9.8 to <11.2 g/d), and Q4 (≥11.2 g/d). No. of obs: available observations of the eligible participants. Mean (95%CI) estimated using linear regression models adjusting for age and sex. * *p* < 0.05 compared with reference group (<8.4 g/d). The β with 95%CI estimated using linear mixed-effects models adjusted for age, sex, residential area, educational level, physical exercise, smoking status, alcohol drinking, and dietary preferences, total cholesterol, triglyceride, high-density lipoprotein cholesterol, low-density lipoprotein cholesterol, fasting blood glucose, hypertension, and diabetes. *p* values: p for interaction between 24 h salt intake and sex.

**Table 3:** Mediating effect of body fat mass on the association between 24 h salt intake and adiposity indices.

	Total Effect	Direct Effect	Indirect Effect	Proportion Mediated (%)
β with 95%CI *				
Body mass index (kg/m^2^)	0.15 (0.13, 0.18)	0.07 (0.05, 0.09)	0.09 (0.06, 0.11)	56.93 (47.96, 66.73)
Body roundness index	0.031 (0.021, 0.041)	0.007 (−0.002, 0.016)	0.024 (0.019, 0.030)	78.57 (58.90, 111.20)
OR with 95%CI *				
Overweight	1.22 (1.15, 1.30)	1.08 (1.03, 1.13)	1.13 (1.10, 1.17)	63.29 (50.38, 83.26)
Central obesity	1.10 (1.04, 1.15)	1.01 (0.97, 1.06)	1.08 (1.06, 1.10)	84.73 (56.17, 171.90)

Abbreviations: CI, confidence interval; OR, odds ratio. Total effect was estimated using linear models (continuous dependent variables) or generalized linear models (binary dependent variables) adjusted for age, sex, residential area, educational level, physical exercise, smoking status, alcohol drinking, and dietary preferences, total cholesterol, triglyceride, high-density lipoprotein cholesterol, low-density lipoprotein cholesterol, fasting blood glucose, hypertension, diabetes, baseline body measurements, and baseline 24 h salt intake; indirect effect and proportion mediated was estimated additionally adjusted for body fat mass measured at baseline and follow-up survey. * The bootstrapped CIs were obtained using 1000 replicates.

## Data Availability

The data presented in this study are available from the corresponding author on reasonable request due to ethical restrictions.

## Supplementary Materials

The following supporting information can be downloaded at https://www.mdpi.com/article/10.3390/nu18060976/s1, Table S1: Association of 24 h salt intake with measures of adiposity using different models; Table S2: Association (β with 95%CI) between 24 h salt intake and measures of adiposity by age groups; Table S3: Association (OR with 95%CI) between 24 h salt intake and the risk of overweight and central obesity by age groups; Table S4: Longitudinal association (β with 95%CI) between 24 h salt intake and measures of adiposity over a 4-year period by age groups; Table S5: Longitudinal association (OR with 95%CI) between 24 h salt intake and the risk of overweight and central obesity over a 4-year period by age groups; Table S6: Association (β with 95%CI) between 24 h salt intake and measures of adiposity among participants with hypertension or diabetes; Table S7: Association (OR with 95%CI) between 24 h salt intake and the risk of overweight and central obesity among participants with hypertension or diabetes; Table S8: Longitudinal association (β with 95%CI) between 24 h salt intake and measures of adiposity over a 4-year period among participants with hypertension or diabetes; Table S9: Longitudinal association (OR with 95%CI) between 24 h salt intake and the risk of overweight and central obesity over a 4-year period among participants with hypertension or diabetes; Figure S1: Flow chart for the selection of study participants.

## Abbreviations

The following abbreviations are used in this manuscript:

BMI	Body mass index
BRI	Body roundness index
95%CI	95% confidence intervals
HDL-c	High-density lipoprotein cholesterol
LDL-c	Low-density lipoprotein cholesterol
LMMs	Linear mixed-effects models
RCTs	Randomized controlled trials
SRHPP	Salt Reduction and Hypertension Prevention Project
TC	Total cholesterol
TG	Triglyceride
ORs	Odds ratios
24-hUNa_pre_	Predicted 24 h urinary sodium excretion
