# Supplementary material for: Association Between Salt Intake and Body Adiposity in Chinese Population: A Repeated-Measures Cohort Study
Source: Nutrients. 2026 Mar 19;18(6):976. doi: 10.3390/nu18060976 (PMC13028953; doi:10.3390/nu18060976)
Supplement: Supplementary file 1 [file nutrients-18-00976-s001.zip › nutrients-4152125-supplementary.pdf]

Table S1 Association of 24-hour salt intake with measures of adiposity using different models

	No. of observations	Adjusted β (95%CI)			
		**Model 1**	**Model 2**	**Model 3**	**Model 4**
**BMI (kg/m^2)**					
**24-hour salt intake [1]**					
<8.4 g/d	3188	Ref	Ref	Ref	Ref
8.4-9.8 g/d	3275	0.35 (0.24, 0.45)	0.34 (0.24, 0.44)	0.33 (0.23, 0.43)	0.31 (0.21, 0.41)
9.8-11.2 g/d	3159	0.72 (0.61, 0.82)	0.71 (0.60, 0.82)	0.70 (0.59, 0.81)	0.69 (0.59, 0.80)
≥11.2 g/d	3178	1.15 (1.03, 1.26)	1.15 (1.03, 1.26)	1.14 (1.02, 1.25)	1.14 (1.03, 1.25)
**BRI**					
**24-hour salt intake [1]**					
<8.4 g/d	3188	Ref	Ref	Ref	Ref
8.4-9.8 g/d	3275	0.10 (0.06, 0.14)	0.10 (0.06, 0.14)	0.09 (0.06, 0.13)	0.08 (0.04, 0.12)
9.8-11.2 g/d	3159	0.20 (0.16, 0.24)	0.20 (0.16, 0.24)	0.19 (0.15, 0.24)	0.19 (0.14, 0.23)
≥11.2 g/d	3178	0.32 (0.28, 0.37)	0.32 (0.28, 0.37)	0.32 (0.28, 0.36)	0.31 (0.26, 0.35)
**Body fat mass (kg)**					
**24-hour salt intake [1]**					
<8.4 g/d	3188	Ref	Ref	Ref	Ref
8.4-9.8 g/d	3275	0.56 (0.38, 0.74)	0.56 (0.38, 0.74)	0.54 (0.36, 0.72)	0.48 (0.31, 0.66)
9.8-11.2 g/d	3159	1.15 (0.96, 1.34)	1.14 (0.95, 1.33)	1.12 (0.93, 1.31)	1.09 (0.91, 1.28)
≥11.2 g/d	3178	1.94 (1.74, 2.14)	1.94 (1.74, 2.14)	1.91 (1.71, 2.11)	1.88 (1.69, 2.07)

[1] **The 24-hour salt intake** was calculated based on the 24-hour urinary Na excretion estimated using spot urine sample with the "Zhejiang model". Participants were then stratified into quartiles based on 24-hour salt intake levels, categorized as Q1(<8.4 g/d), Q2 (8.4 to <9.8 g/d), Q3 (9.8 to <11.2 g/d), and Q4 (≥11.2 g/d).

Model 1: linear mixed effects models adjusted for age and sex.

Model 2: additionally adjusted for residential area and educational level based on model 1.

Model 3: additionally adjusted for physical exercise, smoking status, alcohol drinking, and dietary preferences based on model 2.

Model 4: additionally adjusted for total cholesterol, triglyceride, high-density lipoprotein cholesterol, low-density lipoprotein cholesterol, fasting blood glucose, hypertension, and diabetes based on model 3.

Table S2 Association (β with 95%CI) between 24-hour salt intake and measures of adiposity by age groups

	No. of obs	24-hour salt intake [1]				*P* for interaction
		<8.4 g/d	8.4-9.8 g/d	9.8-11.2 g/d	≥11.2 g/d	
**BMI (kg/m^2)**						
**Age groups (years)**						*0.003*
< 30	1786	Ref	0.29 (-0.10, 0.68)	0.84 (0.44, 1.24)	1.55 (1.15, 1.95)	
30-40	2508	Ref	0.35 (0.03, 0.67)	0.91 (0.60, 1.23)	1.51 (1.20, 1.82)	
40-50	2485	Ref	0.33 (0.05, 0.61)	0.71 (0.43, 0.98)	1.16 (0.88, 1.44)	
50-60	2863	Ref	0.18 (-0.05, 0.40)	0.68 (0.44, 0.92)	1.21 (0.95, 1.47)	
≥ 60	3158	Ref	0.34 (0.19, 0.49)	0.62 (0.42, 0.81)	0.66 (0.41, 0.92)	
**BRI**						
**Age groups (years)**						*0.003*
< 30	1786	Ref	0.08 (-0.04, 0.20)	0.20 (0.08, 0.33)	0.36 (0.24, 0.48)	
30-40	2508	Ref	0.14 (0.03, 0.25)	0.27 (0.17, 0.38)	0.41 (0.30, 0.51)	
40-50	2485	Ref	0.09 (-0.02, 0.19)	0.23 (0.12, 0.33)	0.34 (0.23, 0.44)	
50-60	2863	Ref	0.09 (0.01, 0.18)	0.19 (0.10, 0.28)	0.39 (0.30, 0.49)	
≥ 60	3158	Ref	0.07 (0.00, 0.14)	0.19 (0.10, 0.28)	0.17 (0.05, 0.29)	
**Body fat mass (kg)**						
**Age groups (years)**						*0.008*
< 30	1786	Ref	0.39 (-0.22, 1.00)	1.24 (0.61, 1.87)	2.45 (1.83, 3.07)	
30-40	2508	Ref	0.60 (0.06, 1.14)	1.51 (0.98, 2.04)	2.44 (1.92, 2.97)	
40-50	2485	Ref	0.64 (0.14, 1.13)	1.43 (0.95, 1.92)	2.29 (1.79, 2.78)	
50-60	2863	Ref	0.22 (-0.16, 0.59)	0.93 (0.53, 1.32)	1.86 (1.44, 2.29)	
≥ 60	3158	Ref	0.55 (0.27, 0.84)	1.15 (0.79, 1.51)	1.15 (0.68, 1.62)	

[1] **The 24-hour salt intake** was calculated based on the 24-hour urinary Na excretion estimated using spot urine sample with the "Zhejiang model". Participants were then stratified into quartiles based on 24-hour salt intake levels, categorized as Q1(<8.4 g/d), Q2 (8.4 to <9.8 g/d), Q3 (9.8 to <11.2 g/d), and Q4 (≥11.2 g/d).

No. of obs: available observations of the eligible participants.

The β with 95%CI estimated using linear mixed effects models adjusted for age, sex, residential area, educational level, physical exercise, smoking status, alcohol drinking, and dietary preferences, total cholesterol, triglyceride, high-density lipoprotein cholesterol, low-density lipoprotein cholesterol, fasting blood glucose, hypertension, and diabetes.

***P* for interaction** between 24-hour salt intake and age.

Table S3 Association (OR with 95%CI) between 24-hour salt intake and the risk of overweight and central obesity by age groups

	No. of obs	24-hour salt intake [1]				*P* for interaction
		<8.4 g/d	8.4-9.8 g/d	9.8-11.2 g/d	≥11.2 g/d	
**Overweight**						
**Age groups (years)**						*0.261*
< 30	1786	Ref	1.25 (0.83, 1.89)	1.88 (1.26, 2.82)	3.10 (2.12, 4.52)	
30-40	2508	Ref	1.52 (1.07, 2.16)	2.61 (1.85, 3.68)	3.62 (2.60, 5.05)	
40-50	2485	Ref	1.32 (0.96, 1.83)	1.81 (1.32, 2.47)	2.64 (1.93, 3.61)	
50-60	2863	Ref	1.21 (0.94, 1.55)	1.86 (1.44, 2.40)	3.18 (2.41, 4.19)	
≥ 60	3158	Ref	1.38 (1.13, 1.69)	1.88 (1.46, 2.41)	1.88 (1.36, 2.59)	
**Central obesity**						
**Age groups (years)**						*0.004*
< 30	1786	Ref	1.69 (0.95, 3.01)	2.34 (1.33, 4.09)	3.57 (2.11, 6.04)	
30-40	2508	Ref	1.72 (1.08, 2.75)	2.55 (1.63, 3.99)	3.97 (2.59, 6.09)	
40-50	2485	Ref	1.54 (1.05, 2.25)	1.86 (1.29, 2.69)	3.04 (2.13, 4.35)	
50-60	2863	Ref	1.42 (1.08, 1.87)	2.12 (1.61, 2.78)	3.91 (2.96, 5.18)	
≥ 60	3158	Ref	1.33 (1.09, 1.63)	1.63 (1.28, 2.07)	1.88 (1.38, 2.56)	

[1] **The 24-hour salt intake** was calculated based on the 24-hour urinary Na excretion estimated using spot urine sample with the “Zhejiang model”. Participants were then stratified into quartiles based on 24-hour salt intake levels, categorized as Q1(<8.4 g/d), Q2 (8.4 to <9.8 g/d), Q3 (9.8 to <11.2 g/d), and Q4 (≥11.2 g/d).

No. of obs: available observations of the eligible participants.

The OR with 95%CI estimated using generalized linear mixed effects models adjusted for age, sex, residential area, educational level, physical exercise, smoking status, alcohol drinking, and dietary preferences, total cholesterol, triglyceride, high-density lipoprotein cholesterol, low-density lipoprotein cholesterol, fasting blood glucose, hypertension, and diabetes.

***P* for interaction** between 24-hour salt intake and age.

Table S4 Longitudinal association (β with 95%CI) between 24-hour salt intake and measures of adiposity over a 4-year period by age groups

	No. of sub	24-hour salt intake [1]			*P* for interaction
		Stable	Decrease	Increase	
**BMI (kg/m^2)**					
**Age groups (years)**					*0.019*
< 30	808	Ref	-0.20 (-0.64, 0.25)	**0.48 (0.09, 0.87)**	
30-40	1026	Ref	-0.17 (-0.46, 0.12)	**0.27 (0.01, 0.54)**	
40-50	1146	Ref	-0.15 (-0.40, 0.10)	**0.39 (0.14, 0.63)**	
50-60	1224	Ref	-0.16 (-0.39, 0.06)	**0.32 (0.08, 0.56)**	
≥ 60	1224	Ref	**-0.32 (-0.52, -0.12)**	-0.03 (-0.28, 0.23)	
**BRI**					
**Age groups (years)**					*0.023*
< 30	808	Ref	-0.04 (-0.18, 0.11)	0.12 (-0.01, 0.25)	
30-40	1026	Ref	0.00 (-0.10, 0.11)	**0.13 (0.03, 0.23)**	
40-50	1146	Ref	-0.06 (-0.15, 0.04)	**0.15 (0.06, 0.24)**	
50-60	1224	Ref	-0.02 (-0.11, 0.08)	**0.12 (0.02, 0.22)**	
≥ 60	1224	Ref	-0.08 (-0.18, 0.01)	-0.03 (-0.16, 0.09)	
**Body fat mass (kg)**					
**Age groups (years)**					*0.007*
< 30	808	Ref	**-0.72 (-1.43, -0.01)**	0.52 (-0.11, 1.14)	
30-40	1026	Ref	-0.29 (-0.82, 0.25)	0.09 (-0.41, 0.58)	
40-50	1146	Ref	0.22 (-0.22, 0.66)	**0.89 (0.46, 1.32)**	
50-60	1224	Ref	0.31 (-0.11, 0.73)	0.38 (-0.07, 0.83)	
≥ 60	1224	Ref	**-0.42 (-0.81, -0.03)**	-0.40 (-0.90, 0.10)	

[1] **The 24-hour salt intake** was calculated based on the 24-hour urinary Na excretion estimated using spot urine sample with the "Zhejiang model". Participants were divided into 3 groups: **stable** (change in 24-hour salt intake <1g between baseline and follow-up), **decrease** (follow-up 24-hour salt intake was 1g lower than baseline), and **increase** (follow-up 24-hour salt intake was 1g higher than baseline).

No. of subs: number of eligible subjects participated in both baseline and follow-up survey.

The β with 95%CI estimated using linear models adjusted for baseline age, sex, residential area, educational level, physical exercise, smoking status, alcohol drinking, and dietary preferences, total cholesterol, triglyceride, high-density lipoprotein cholesterol, low-density lipoprotein cholesterol, fasting blood glucose, hypertension, diabetes, and baseline body measurements.

***P* for interaction** between 24-hour salt intake group and age.

Table S5 Longitudinal association (OR with 95%CI) between 24-hour salt intake and the risk of overweight and central obesity over a 4-year period by age groups

	No. of sub	24-hour salt intake [1]			*P* for interaction
		Stable	Decrease	Increase	
**Overweight**					
**Age groups (years)**					*0.379*
< 30	808	Ref	1.37 (0.81, 2.32)	**1.64 (1.03, 2.61)**	
30-40	1026	Ref	**0.52 (0.33, 0.84)**	1.07 (0.70, 1.64)	
40-50	1146	Ref	0.88 (0.57, 1.35)	**1.55 (1.02, 2.35)**	
50-60	1224	Ref	0.99 (0.66, 1.50)	**1.64 (1.07, 2.54)**	
≥ 60	1224	Ref	0.79 (0.53, 1.17)	1.36 (0.83, 2.23)	
**Central obesity**					
**Age groups (years)**					*0.013*
< 30	808	Ref	0.93 (0.52, 1.68)	1.39 (0.82, 2.35)	
30-40	1026	Ref	1.02 (0.63, 1.66)	**1.79 (1.14, 2.81)**	
40-50	1146	Ref	1.01 (0.66, 1.54)	1.45 (0.96, 2.19)	
50-60	1224	Ref	0.77 (0.54, 1.10)	1.24 (0.85, 1.82)	
≥ 60	1224	Ref	0.85 (0.61, 1.18)	0.85 (0.55, 1.32)	

[1] **The 24-hour salt intake** was calculated based on the 24-hour urinary Na excretion estimated using spot urine sample with the "Zhejiang model". Participants were divided into 3 groups: **stable** (change in 24-hour salt intake <1g between baseline and follow-up), **decrease** (follow-up 24-hour salt intake was 1g lower than baseline), and **increase** (follow-up 24-hour salt intake was 1g higher than baseline).

No. of subs: number of eligible subjects participated in both baseline and follow-up survey.

The OR with 95%CI estimated using generalized linear models adjusted for baseline age, sex, residential area, educational level, physical exercise, smoking status, alcohol drinking, and dietary preferences, total cholesterol, triglyceride, high-density lipoprotein cholesterol, low-density lipoprotein cholesterol, fasting blood glucose, hypertension, diabetes, and baseline body measurements.

P for interaction between 24-hour salt intake group and age.

Table S6 Association (β with 95%CI) between 24-hour salt intake and measures of adiposity among participants with hypertension or diabetes

	No. of obs	24-hour salt intake [1]				*P* for interaction
		<8.4 g/d	8.4-9.8 g/d	9.8-11.2 g/d	≥11.2 g/d	
**BMI (kg/m^2)**						
**Health status**						*0.107*
None	7762	Ref	0.33 (0.19, 0.47)	0.79 (0.65, 0.93)	1.29 (1.14, 1.44)	
Diabetes	350	Ref	0.71 (-0.05, 1.47)	1.08 (0.31, 1.85)	1.63 (0.78, 2.48)	
Hypertension	3885	Ref	0.23 (0.05, 0.40)	0.52 (0.33, 0.72)	1.03 (0.81, 1.24)	
Both	803	Ref	0.42 (0.05, 0.80)	0.45 (0.03, 0.87)	0.76 (0.31, 1.21)	
**BRI**						
**Health status**						*0.124*
None	7762	Ref	0.08 (0.03, 0.13)	0.21 (0.16, 0.26)	0.33 (0.28, 0.39)	
Diabetes	350	Ref	0.16 (-0.12, 0.44)	0.38 (0.10, 0.67)	0.50 (0.19, 0.80)	
Hypertension	3885	Ref	0.06 (-0.01, 0.14)	0.16 (0.09, 0.24)	0.31 (0.23, 0.40)	
Both	803	Ref	0.12 (-0.04, 0.29)	0.10 (-0.08, 0.29)	0.20 (0.01, 0.40)	
**Body fat mass (kg)**						
**Health status**						*0.038*
None	7762	Ref	0.63 (0.39, 0.86)	1.30 (1.06, 1.54)	2.19 (1.94, 2.45)	
Diabetes	350	Ref	0.13 (-1.11, 1.37)	1.49 (0.22, 2.75)	2.91 (1.55, 4.27)	
Hypertension	3885	Ref	0.28 (-0.03, 0.59)	0.88 (0.54, 1.22)	1.86 (1.48, 2.23)	
Both	803	Ref	0.33 (-0.35, 1.01)	0.52 (-0.23, 1.27)	0.86 (0.05, 1.66)	

[1] **The 24-hour salt intake** was calculated based on the 24-hour urinary Na excretion estimated using spot urine sample with the “Zhejiang model”. Participants were then stratified into quartiles based on 24-hour salt intake levels, categorized as Q1(<8.4 g/d), Q2 (8.4 to <9.8 g/d), Q3 (9.8 to <11.2 g/d), and Q4 (≥11.2 g/d).

No. of obs: available observations of the eligible participants.

Health status: participants were categorized into four groups: no chronic disease, diabetes only, hypertension only, and both hypertension and diabetes.

The β with 95%CI estimated using linear mixed effects models adjusted for age, sex, residential area, educational level, physical exercise, smoking status, alcohol drinking, and dietary preferences, total cholesterol, triglyceride, high-density lipoprotein cholesterol, and low-density lipoprotein cholesterol.

***P* for interaction** between 24-hour salt intake and health status.

Table S7 Association (OR with 95%CI) between 24-hour salt intake and the risk of overweight and central obesity among participants with hypertension or diabetes

	No. of obs	24-hour salt intake [1]				*P* for interaction
		<8.4 g/d	8.4-9.8 g/d	9.8-11.2 g/d	≥11.2 g/d	
**Overweight**						
**Health status**						*0.273*
None	7762	Ref	1.36 (1.15, 1.61)	2.22 (1.87, 2.63)	3.08 (2.59, 3.65)	
Diabetes	350	Ref	2.11 (1.02, 4.34)	3.13 (1.47, 6.66)	4.23 (1.93, 9.30)	
Hypertension	3885	Ref	1.36 (1.11, 1.67)	1.74 (1.39, 2.17)	2.71 (2.12, 3.46)	
Both	803	Ref	1.28 (0.80, 2.06)	1.42 (0.84, 2.39)	2.00 (1.13, 3.54)	
**Central obesity**						
**Health status**						*0.206*
None	7762	Ref	1.43 (1.16, 1.76)	2.02 (1.64, 2.48)	3.02 (2.46, 3.71)	
Diabetes	350	Ref	1.76 (0.83, 3.73)	3.04 (1.39, 6.64)	4.66 (2.05, 10.59)	
Hypertension	3885	Ref	1.35 (1.10, 1.65)	1.68 (1.35, 2.09)	2.99 (2.37, 3.76)	
Both	803	Ref	1.36 (0.89, 2.07)	1.29 (0.82, 2.04)	1.80 (1.11, 2.91)	

[1] **The 24-hour salt intake** was calculated based on the 24-hour urinary Na excretion estimated using spot urine sample with the "Zhejiang model". Participants were then stratified into quartiles based on 24-hour salt intake levels, categorized as Q1(<8.4 g/d), Q2 (8.4 to <9.8 g/d), Q3 (9.8 to <11.2 g/d), and Q4 (≥11.2 g/d).

No. of obs: available observations of the eligible participants.

Health status: participants were categorized into four groups: no chronic disease, diabetes only, hypertension only, and both hypertension and diabetes.

The OR with 95%CI estimated using generalized linear mixed effects models adjusted for age, sex, residential area, educational level, physical exercise, smoking status, alcohol drinking, and dietary preferences, total cholesterol, triglyceride, high-density lipoprotein cholesterol, and low-density lipoprotein cholesterol.

***P* for interaction** between 24-hour salt intake and health status.

Table S8 Longitudinal association (β with 95%CI) between 24-hour salt intake and measures of adiposity over a 4-year period among participants with hypertension or diabetes

	No. of sub	24-hour salt intake [1]			*P* for interaction
		Stable	Decrease	Increase	
**BMI (kg/m^2)**					
**Health status**					*0.126*
None	3252	Ref	**-0.19 (-0.35, -0.03)**	**0.35 (0.19, 0.50)**	
Diabetes	123	Ref	-0.24 (-0.92, 0.43)	-0.52 (-1.29, 0.26)	
Hypertension	1750	Ref	**-0.23 (-0.42, -0.03)**	0.15 (-0.07, 0.37)	
Both	303	Ref	-0.15 (-0.56, 0.26)	0.29 (-0.16, 0.73)	
**BRI**					
**Health status**					*0.202*
None	3252	Ref	-0.04 (-0.10, 0.02)	**0.11 (0.05, 0.17)**	
Diabetes	123	Ref	-0.10 (-0.36, 0.15)	-0.30 (-0.59, 0.00)	
Hypertension	1750	Ref	-0.05 (-0.13, 0.03)	0.06 (-0.03, 0.15)	
Both	303	Ref	0.03 (-0.17, 0.22)	0.04 (-0.17, 0.25)	
**Body fat mass (kg)**					
**Health status**					*0.026*
None	3252	Ref	-0.23 (-0.52, 0.06)	**0.41 (0.13, 0.69)**	
Diabetes	123	Ref	0.51 (-0.71, 1.72)	0.00 (-1.38, 1.38)	
Hypertension	1750	Ref	-0.03 (-0.39, 0.33)	0.22 (-0.17, 0.62)	
Both	303	Ref	-0.18 (-0.99, 0.63)	-0.47 (-1.35, 0.41)	

[1] **The 24-hour salt intake** was calculated based on the 24-hour urinary Na excretion estimated using spot urine sample with the "Zhejiang model". Participants were divided into 3 groups: **stable** (change in 24-hour salt intake <1g between baseline and follow-up), **decrease** (follow-up 24-hour salt intake was 1g lower than baseline), and **increase** (follow-up 24-hour salt intake was 1g higher than baseline).

No. of sub: number of eligible subjects participated in both baseline and follow-up survey.

Health status: participants were categorized into four groups based on their baseline heath status: no chronic disease, diabetes only, hypertension only, and both hypertension and diabetes.

The β with 95%CI estimated using linear models adjusted for baseline age, sex, residential area, educational level, physical exercise, smoking status, alcohol drinking, and dietary preferences, total cholesterol, triglyceride, high-density lipoprotein cholesterol, low-density lipoprotein cholesterol, and baseline body measurements.

***P* for interaction** between 24-hour salt intake group and health status.

Table S9 Longitudinal association (OR with 95%CI) between 24-hour salt intake and the risk of overweight and central obesity over a 4-year period among participants with hypertension or diabetes

	No. of sub	24-hour salt intake [1]			*P* for interaction
		Stable	Decrease	Increase	
**Overweight**					
**Health status**					*0.148*
None	3252	Ref	0.96 (0.75, 1.23)	**1.54 (1.22, 1.94)**	
Diabetes	123	Ref	1.54 (0.41, 5.78)	0.74 (0.17, 3.22)	
Hypertension	1750	Ref	**0.65 (0.46, 0.90)**	1.03 (0.71, 1.48)	
Both	303	Ref	0.73 (0.31, 1.72)	1.48 (0.57, 3.81)	
**Central obesity**					
**Health status**					*0.436*
None	3252	Ref	0.83 (0.65, 1.06)	**1.27 (1.00, 1.62)**	
Diabetes	123	Ref	1.35 (0.38, 4.78)	0.31 (0.07, 1.40)	
Hypertension	1750	Ref	0.85 (0.63, 1.13)	1.15 (0.83, 1.59)	
Both	303	Ref	1.28 (0.61, 2.70)	1.32 (0.57, 3.07)	

[1] **The 24-hour salt intake** was calculated based on the 24-hour urinary Na excretion estimated using spot urine sample with the "Zhejiang model". Participants were divided into 3 groups: **stable** (change in 24-hour salt intake <1g between baseline and follow-up), **decrease** (follow-up 24-hour salt intake was 1g lower than baseline), and **increase** (follow-up 24-hour salt intake was 1g higher than baseline).

No. of sub: number of eligible subjects participated in both baseline and follow-up survey.

Health status: participants were categorized into four groups based on their baseline heath status: no chronic disease, diabetes only, hypertension only, and both hypertension and diabetes.

The OR with 95%CI estimated using generalized linear models adjusted for baseline age, sex, residential area, educational level, physical exercise, smoking status, alcohol drinking, and dietary preferences, total cholesterol, triglyceride, high-density lipoprotein cholesterol, low-density lipoprotein cholesterol, and baseline body measurements.

***P* for interaction** between 24-hour salt intake group and health status.

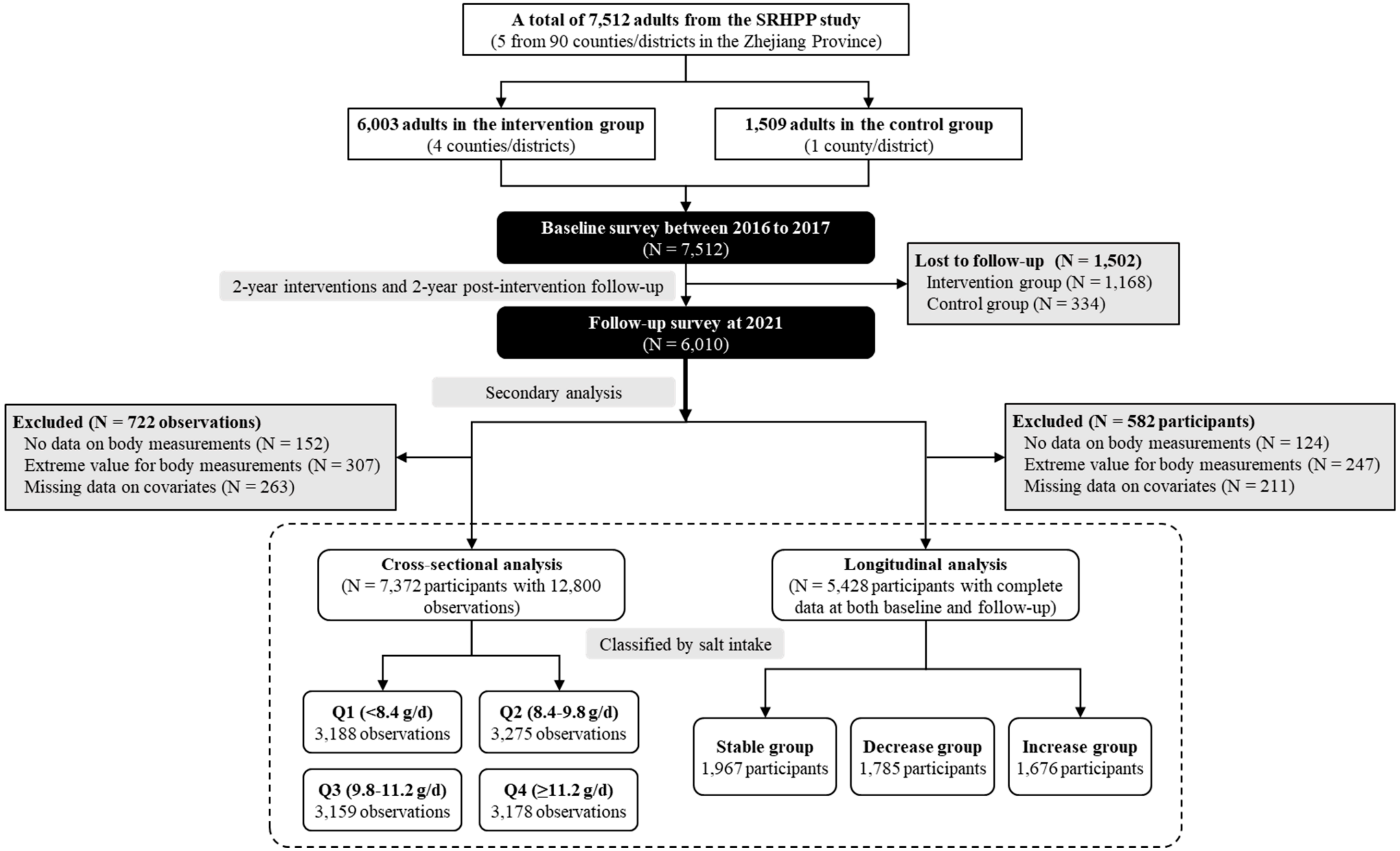

Figure S1 Flow chart for the selection of study participants

The 24-hour salt intake was calculated based on the 24-hour urinary Na excretion estimated using spot urine sample with the "Zhejiang model".

Cross-sectional analysis: participants were then stratified into quartiles based on 24-hour salt intake levels, categorized as Q1(<8.4 g/d), Q2 (8.4 to <9.8 g/d), Q3 (9.8 to <11.2 g/d), and Q4 (≥11.2 g/d).

Longitudinal analysis: participants were divided into 3 groups: **stable** (change in 24-hour salt intake <1g between baseline and follow-up), **decrease** (follow-up 24-hour salt intake was 1g lower than baseline), and **increase** (follow-up 24-hour salt intake was 1g higher than baseline).
